# Comparative study of a reference PCR assay targeting kinetoplast DNA and a commercial kit targeting ribosomal DNA for the molecular diagnosis of visceral leishmaniasis

**DOI:** 10.1128/jcm.00666-26

**Published:** 2026-09-25

**Authors:** Quentin Andreotti, Eva Lemont, Nada Kuk, Mathilde Criado, Christophe Ravel, Yvon Sterkers

**Affiliations:** 1 Department of Parasitology-Mycology, National Reference Center for Leishmaniasis, University of Montpellier, CNRS, IRD, University Hospital Center (CHU) of Montpellier, MiVEGEC 27037, Montpellier, France; Mayo Clinic Minnesota, Rochester, Minnesota, USA

**Keywords:** molecular diagnosis, ribosomal DNA, kinetoplast DNA, quantitative PCR, visceral leishmaniasis

## Abstract

Visceral leishmaniasis (VL) is a severe parasitic disease that requires accurate and timely diagnosis to prevent fatal outcomes. Molecular methods, particularly quantitative PCR (qPCR), have become central to VL diagnosis because of their high sensitivity and specificity. However, assay performance depends on several factors, including the choice of DNA target. We compared the analytical and clinical performance of an in-house reference qPCR targeting kinetoplast DNA with the Viasure *Leishmania* Real-Time PCR Detection Kit targeting 18S ribosomal DNA. Analytical evaluation included PCR efficiency, linearity ranges, and detection limits. Clinical performance was assessed using 30 blood samples representative of routine diagnostic practice. Both assays showed high efficiency and broad linearity, but the kinetoplast DNA assay achieved a lower limit of detection. Clinical comparison performed on a cohort representative of the routine activity revealed substantial agreement, although the Viasure method produced several false-negative results and non-reproducible positives (amplification in only one of two replicate wells), mainly in samples with very low parasite levels. This likely reflected differences in target copy number, as kinetoplast DNA is present in thousands of copies per parasite, whereas ribosomal DNA occurs in only a few dozen copies. High analytical sensitivity is essential for diagnosis and follow-up because minimal parasitemia is common, and missed diagnoses can have severe consequences. Our findings indicate that assays targeting kinetoplast DNA should be preferred over ribosomal DNA-based alternatives for blood-based diagnosis and monitoring of visceral leishmaniasis.

**IMPORTANCE:**

Accurate diagnosis of visceral leishmaniasis (VL) is essential to prevent delayed treatment and fatal outcomes. Molecular assays have become central to diagnosis, but their performance varies with the genetic target used. This study shows that assays targeting kinetoplast DNA provide superior sensitivity compared to ribosomal DNA-based methods, particularly in cases with very low parasite burdens—a common scenario in clinical practice. The difference likely reflects the much higher copy number of kinetoplast DNA, which enhances detection when parasite levels approach the limit of detection. These findings underscore the need for assay developers to prioritize high-copy-number targets to ensure reliable diagnosis and effective monitoring of treatment response, reducing the risk of relapse and improving patient outcomes.

## INTRODUCTION

Leishmaniasis is a parasitic vector-borne disease that manifests in different clinical forms, including cutaneous leishmaniasis (CL), mucocutaneous leishmaniasis (ML), and visceral leishmaniasis (VL). It is caused by species of the *Leishmania* genus, a group of flagellated protozoa transmitted by female dipterans of the Phlebotominae subfamily (1). Among the different clinical forms, VL is the less frequent but the most severe, being potentially lethal if appropriate treatment is not administered. The World Health Organization classifies VL as a neglected tropical disease and emphasizes the need for control and prevention, particularly through early diagnosis, prompt, effective treatment, and vector control (2). Incidence in 2023 was 50,000–90,000 reported cases (1–3). VL is caused by *L. infantum* in the Mediterranean Basin, the Middle East, Central and South America, and *L. donovani* in Asia and Africa. The two species are phylogenetically very close and constitute the *Leishmania donovani* complex (4). The treatments are not significantly different; therefore, unlike in CL or ML, precise species identification is not required for the clinical and therapeutic management of VL (5, 6).

The diagnosis of VL can be quite challenging, as its clinical symptoms are not specific (7, 8). Detection is further complicated by the parasites’ tendency to preferentially localize the bone marrow, spleen, and lymph nodes, which hampers their detection in blood (9). Direct visualization of parasites through microscopy or culture has traditionally been considered the gold standard for diagnosing VL, primarily because of its high specificity (7, 10). However, its sensitivity is variable and largely depends on the type of sample analyzed (in the case of VL, ranging from spleen aspiration to bone marrow aspiration, lymph node aspiration, or peripheral blood), with reported values between 53% and 99% (10). Moreover, the requirement for specialized expertise in morphological identification, coupled with the inherently prolonged nature of culture-based procedures, has contributed to a progressive decline in their use for routine clinical diagnostic applications (7, 10, 11). Serological diagnostic methods are also employed, based on the detection of specific humoral immune responses (8). These include K39 rapid diagnostic test (RDT), indirect fluorescent antibody test (IFAT), enzyme-linked immunosorbent assay (ELISA), immunoblotting, direct agglutination test (DAT), immunochromatographic tests (ICTs), and antigen detection assays (7, 8, 10, 12, 13). Immunological and serological tests remain the most widely used in endemic areas, largely because sophisticated methods are difficult to implement, while these tests are simple, rapid, and well-suited for field conditions (8, 11). However, these techniques present limitations; they cannot distinguish asymptomatic infections from active clinical cases, and the persistence of antibodies after treatment complicates follow-up and therapeutic management (7, 10).

Molecular techniques, particularly PCR, have revolutionized the diagnosis of infectious diseases by allowing rapid and highly sensitive and specific detection of pathogen DNA or RNA (14) and enabling quantification and load monitoring for patient follow-up (11, 14–16). Many laboratories have developed and implemented their own laboratory-developed PCR assays for VL. Among the most frequently used amplification targets are the small subunit ribosomal RNA (SSU rRNA) and the kinetoplast DNA minicircle (17–19). Kinetoplast DNA represents the unique mitochondrial DNA of kinetoplastid protozoa. It consists of two molecular forms, minicircles and maxicircles, concatenated within a single large DNA network located near the basal body of the flagellum. Each cell contains about 20–50 copies of maxicircles (20–40 kb in size), which encode components of the respiratory chain, and approximately 5,000–10,000 copies of minicircles (0.5–1 kb), which play a key role in regulating maxicircle gene expression (20). The 18S rRNA gene is transcribed from a multigene family composed of conserved segments interspersed with variable spacer sequences that are subject to low selective pressure (21, 22). These features make it a widely used target for the diagnosis of numerous protozoa and fungi (23–25). In *Leishmania spp*., the rRNA genes are organized on chromosome 27, and the number of copies is estimated to be about 20–40. Comparative studies showed that the kinetoplast DNA target provides greater sensitivity than the rRNA target in the detection of *Leishmania* species (26–29). Several commercial DNA-based diagnosis kits are available for the diagnosis of VL from peripheral blood samples, targeting various DNA sequences (see Table 1).

**TABLE 1:** Non-exhaustive list of commercially available qPCR diagnostic kits for detection of *L. donovani* and *L. infantum*

Product name	Targeted gene	Reported sensitivity	Species detected
Viasure *Leishmania* Real-Time PCR Detection Kit	18S rRNA	10 copies/PCR	Pan-*Leishmania*
quanty *Leishmania* Clonit	18S rRNA	1.38 copies/µL	Pan-*Leishmania*
*Leishmania* genesig Standard Kit Primer Design	Cytochrome b	100 copies/PCR	Pan-*Leishmania*
*Leishmania infantum* and *Leishmania donovani* Advanced Kit MyBioSource^*a*^	DNA pol I protein B	100 copies/PCR	*L. infantum* and *L. donovani*
STAT-NAT *Leishmania* spp. Sentinel Diagnostics^*b*^	Not precised	Not precised	*Leishmania* spp.*^a^*
*Leishmania* spp. Real-TM Sacace	Not precised	1000 copies/mL	Pan-*Leishmania*

^*a*^ Discontinued.

^*b*^ Arnau et al. (28) reported that the STAT-NAT *Leishmania* spp. failed to detect the New World species *L. braziliensis* and *L. panamensis*.

**Fig 1:**
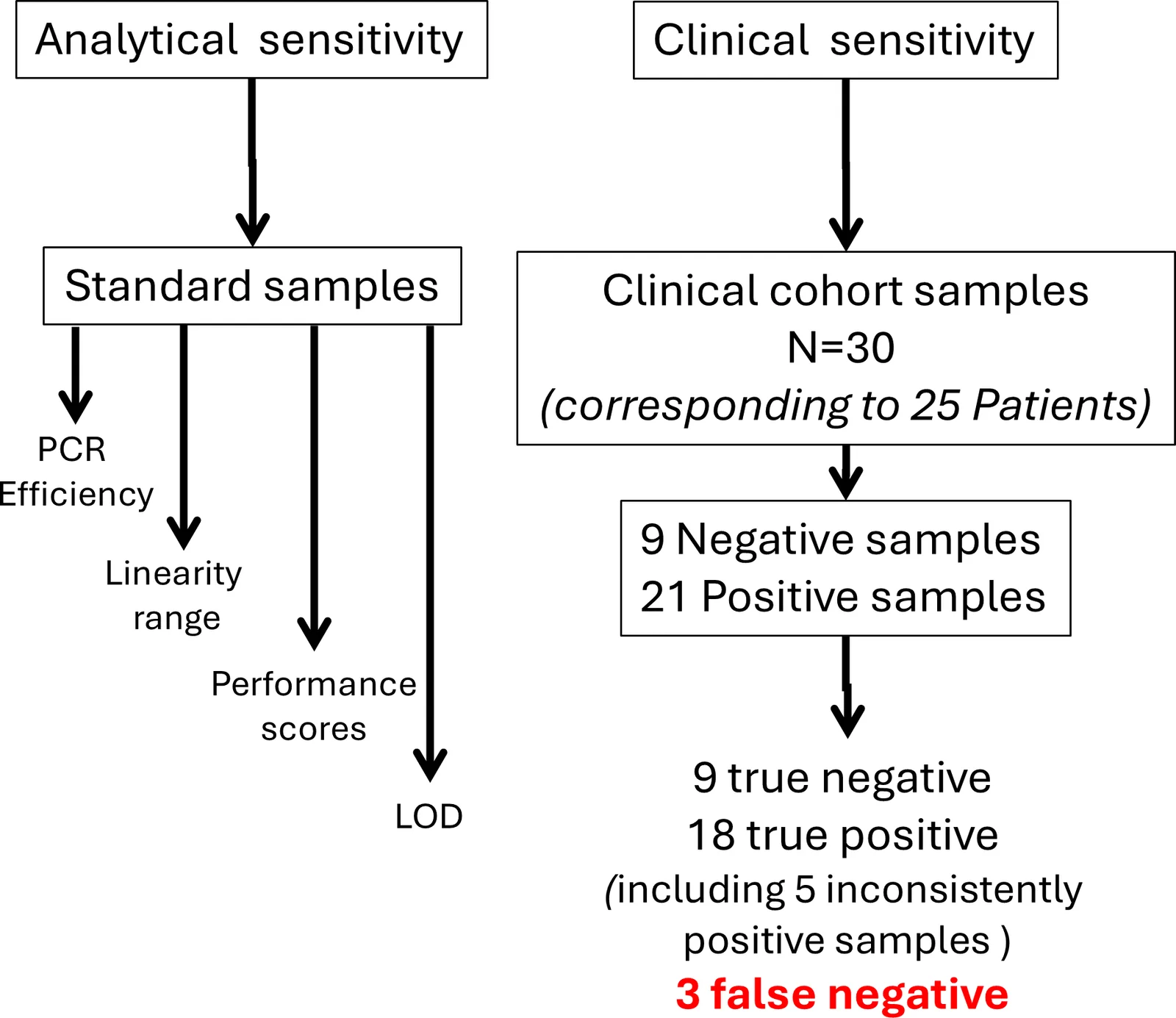
Flowchart of the samples analyzed The comparison study was based on spiked samples obtained from dilution of an *L. infantum* strain to assess analytical sensitivity and a clinical cohort of 25 patients to assess clinical sensitivity.

This study aims to independently evaluate a commercial qPCR kit and determine how the choice of DNA target influences analytical sensitivity, given that the high copy number of kinetoplast DNA relative to ribosomal DNA is expected to enhance detection. We compared and evaluated the performance of an optimized reference method developed by Mary et al. (30) targeting kinetoplast DNA, and the Viasure *Leishmania* Real-Time PCR Detection Kit targeting the 18S ribosomal DNA. We assessed the analytical sensitivity of both methods using spiked samples of a *L. infantum* reference strain. This evaluation allowed determination of PCR efficiency, linearity range, overall performance score, and limit of detection (LOD). We also investigated clinical sensitivity through a cohort of samples representative of the cases encountered in diagnostic practice (Fig. 1). These findings highlight that assay performance is critically dependent on the choice of DNA target, with kinetoplast DNA offering markedly superior sensitivity compared to ribosomal DNA, and emphasize the need for developers to prioritize high-copy-number targets for reliable visceral leishmaniasis diagnosis.

## MATERIALS AND METHODS

Two types of samples were included in this study: standard samples and clinical blood samples (Fig. 1).

### Standard samples

For the evaluation of the analytical sensitivity of the two PCR methods, spiked standard samples were produced. For this purpose, an *L. infantum* strain (MHOM/MA/67/ITMAP263, clone S9F1), hereafter referred to as S9F1, was used. The strain was provided by the French Biological Resource Center for Leishmaniasis in Montpellier of the World Federation for Culture Collection (WDCM 879). Parasites were grown *in vitro* as promastigote forms in HOMEM medium (31). Genomic DNA of S9F1 was extracted using the QIAamp DNA Mini Kit (cat. no. 51306; Qiagen, Courtaboeuf, France) following the manufacturer’s instructions, using 200 µL of parasite culture as input and a final elution volume of 200 µL. To determine PCR efficiency, linearity range, and the performance scores, S9F1 promastigotes were cultured at a concentration of 10^7^ parasites/mL. Genomic DNA was extracted and subjected to 10-fold serial dilutions, which were analyzed in replicate using both PCR methods. To determine the LOD under conditions mimicking clinical samples, *Leishmania*-negative blood samples were spiked with S9F1 promastigotes prior to DNA extraction. Three spiked samples were prepared to yield final concentrations corresponding to 1, 0.5, and 0.25 *Leishmania* per PCR well. For each concentration, 10 µL of parasite culture was added to 200 µL of human blood, and DNA extraction was subsequently performed according to the manufacturer’s protocol.

To assess specificity, (i) procyclic forms of *Trypanosoma brucei brucei*, Lister 427 strain, were cultured at 27°C in SDM-79 (PAA Laboratories) supplemented with heat-inactivated 10% FBS (Gibco) and 7 μg/mL hemin (32), and (ii) positive samples of the QCMD 2025 *Trypanosoma cruzi* (Chagas disease) EQA Pilot Study were included. In both cases, genomic DNA was extracted using the QIAamp DNA Mini Kit as described above.

### Clinical samples

For the evaluation of clinical sensitivity, 30 clinical blood samples were included. These consisted of 21 consecutive positive samples from patients tested for VL at Montpellier University Hospital between February 2020 and August 2022, and 9 negative control samples collected during the same period (Fig. 1; Table S1). DNA from clinical samples was extracted using the QIAamp DNA Mini Kit (cat. no. 51306; Qiagen) from buffy coat samples collected before January 2021. From January 2021 onwards, DNA extraction was performed from whole blood using the MagNA Pure Compact system with the MagNA Pure Compact Nucleic Acid Isolation Kit I (cat. no. 03730964001; Roche, Meylan, France), applying the same input and elution volumes as in the manual extraction method. A definite diagnosis of VL was based on (i) clinical signs and symptoms consistent with VL, (ii) positive *Leishmania* serology or qPCR on another type of specimen, and (iii) a clinically confirmed diagnosis of VL with appropriate treatment. The nine control samples were obtained from patients whose routine PCR tests were negative and in whom VL had been definitively ruled out. The cohort was designed to reflect the cases encountered during the routine activity of the diagnostic laboratory. Accordingly, 13 samples showed a Cq value greater than 30, including 8 samples with a Cq value above 35, corresponding to a parasite load below 10 *Leishmania*/mL of blood (Table S1). Over the past decade, it has been estimated that approximately one-quarter of positive samples represent low-parasite-burden cases (CNR-L, unpublished data).

### PCR methods

The reference method was an in-house qPCR assay developed by Mary et al. (30) targeting a conserved region of the kinetoplast DNA. This assay is the most currently used by the 11 French laboratories that routinely perform molecular diagnosis of VL (29). The amplicon length is approximately 120 bp, and detection is based on a hydrolysis probe (TaqMan) monitored in the HEX channel (533–580 nm). The primer and probe sequences were: RV1-CM-F 5′-CTTTTCTGGTCCTCCGGGTAGG, RV2-CM-R 5′-CCACCCGGCCCTATTTTACACCAA, and minicircle_prob_1 5′-[YAKYE]-TTTTCGCAGAACGCCCCTACCCGC-[ECLIP] (Eurofins Genomics, Ebersberg, Germany). Each reaction consisted of 2 µL of template DNA in a total volume of 20 µL. Amplification was carried out on a LightCycler 480 (Roche) with the following cycling conditions: 45 cycles of denaturation at 95°C for 10 s, followed by annealing/extension at 60°C for 30 s. The assay has been accredited according to ISO 15189 since 2017. All qPCR runs were performed according to good laboratory practice. Reactions were set up in duplicate and included positive control, negative control, and internal control (IC). The positive control consisted of the target sequence, containing the primers and the probe, synthesized and cloned into a pUC vector by IDT (Integrated DNA Technologies), used at a defined concentration corresponding to a quantification cycle (Cq) value of 29.7 ± 0.4. To monitor PCR inhibition, an internal control consisting of positive control was added to the patient DNA sample in a third reaction well. Inhibition was considered absent when the difference between the Cq values of the positive control and the internal control was less than three cycles. In addition, a qPCR assay targeting the albumin gene was performed in a fourth reaction well as a cellularity control. The sequences of the primers and probe for the albumin assay were: Albu-F 5′-GCTGTCATCTCTTGTGGGCTGT, Albu-R 5′-ACTCATGGGAGCTGCTGGTTC, and Albu-Probe 5′-[FAM]-GGAGAGATTTGTGTGGGCATGACA-[BHQ1] (33).

The Viasure *Leishmania* Real-Time PCR Detection Kit (cat. no. VS-LEI196TE; CerTest Biotec, Zaragoza, Spain) is a CE-marked diagnostic kit compliant with the European *in vitro* diagnostic directive 98/79/CE. This multiplex qPCR assay is designed for the qualitative detection of *Leishmania* spp. in various biological samples, including cutaneous biopsies, blood, and bone marrow aspirates, from patients presenting clinical signs of leishmaniasis. The kit targets a conserved region of the 18S ribosomal DNA gene, with detection based on a hydrolysis probe (TaqMan) monitored on the FAM channel (465–510 nm); the primer and probe sequences are proprietary. Each reaction contained 5 µL of template DNA in a total volume of 20 µL, and amplification was performed on a LightCycler 480 (Roche) under the following cycling conditions: 45 cycles of denaturation at 95°C for 10 s, followed by annealing/extension at 60°C for 50 s. The kit contains reagents sufficient for 96 amplification tests, including lyophilized reaction mixes, a rehydration buffer, a lyophilized synthetic non-infectious DNA positive control, a lyophilized non-infectious nucleic acid internal control (IC), a negative control, and RNase/DNase-free water. The IC is detected on the HEX channel (533–580 nm). As part of this study, the analyses were performed in duplicate. For both methods, Cq values were calculated using the absolute quantification/second derivative maxima method (LightCycler 480 software version 1.5.1.62; Roche). The value for each well, as well as the average value, is reported in Table S1.

### Analysis of performance and statistical analysis

From a S9F1 standard DNA sample containing 10^7^ *Leishmania*/mL, nine successive tenfold dilutions were prepared. The four highest concentrations were tested in duplicate, whereas the lowest concentrations were tested in quadruplicate, resulting in a total of 32 reaction wells (Table 2). qPCR performances were evaluated by determining the linear range of the standard curve, and PCR efficiency was calculated from the slope of the curve using the formula [efficiency (EFF) = (−1 + 10^(−1/slope)^) × 100]. To facilitate performance comparison between methods, two scoring metrics were applied as described by Sterkers et al. (34): the “total score/32,” representing the number of positive wells out of 32, and the “most stringent score/12,” calculated from results obtained at the three lowest concentrations. For qualitative results, the agreement was evaluated with Cohen’s kappa coefficient. For quantitative results, agreement and correlation between the two qPCR techniques were assessed using a Bland-Altman plot and a correlation plot. LOD corresponds to the lowest target concentration that can be detected with a statistical confidence level of 95%. To estimate the LOD, three concentrations prepared during DNA extraction (1, 0.5, and 0.25 *Leishmania* per PCR well) were tested 20 times. The qualitative results of the 20 replicates were used to calculate the hit rate for both methods.

**TABLE 2:** Comparison of Cq values of the two qPCR methods (with/without IC for Viasure) obtained in the one-tenth serial dilution assays

	Conc. (Leish./mL)	10^7^	10^6^	10^5^	10^4^	10^3^	10^2^	10	1	0.1	0.01	Score/32	Stringent score/12
In-house qPCR assay developed by Mary et al. (30)	Qualitative result	2/2	2/2	2/2	2/2	4/4	4/4	4/4	4/4	2/4	1/4	27 (84%)	7 (58%)
	Cq value (mean ± SD)^*a*^	15.8	19.3	22.6	25.9	29.5 ± 0.1	32.7 ± 0.4	35.1 ± 0.8	38.9 ± 1.3	39.6	37.9		
“Leishmania Viasure” without IC	Qualitative result	2/2	2/2	2/2	2/2	4/4	4/4	0/4	2/4	0/4	0/4	18 (56%)	2 (17%)
	Cq value (mean ± SD)^*a*^	16.1	19.4	22.8	26.2	29.7 ± 0.1	33.2 ± 0.5	Neg	35.9	Neg	Neg		
“Leishmania Viasure” with IC	Qualitative result	2/2	2/2	2/2	2/2	4/4	4/4	1/4	1/4	0/4	0/4	18 (56%)	1 (8%)
	Cq value (mean ± SD)^*a*^	16.1	19.6	22.9	26.5	30.0 ± 0.2	33.9 ± 0.9	37.3	40	Neg	Neg		

^*a*^ Standard deviations (SDs) were calculated when applicable, that is, when at least three Cq values were available. “Total score/32” refers to the total number of positive wells out of 32 tested. “Most stringent score/12” refers to the number of positive wells in the three lowest concentrations tested.

## RESULTS

### Analytical performances of the Viasure kit compared to the reference method

**Fig 2:**
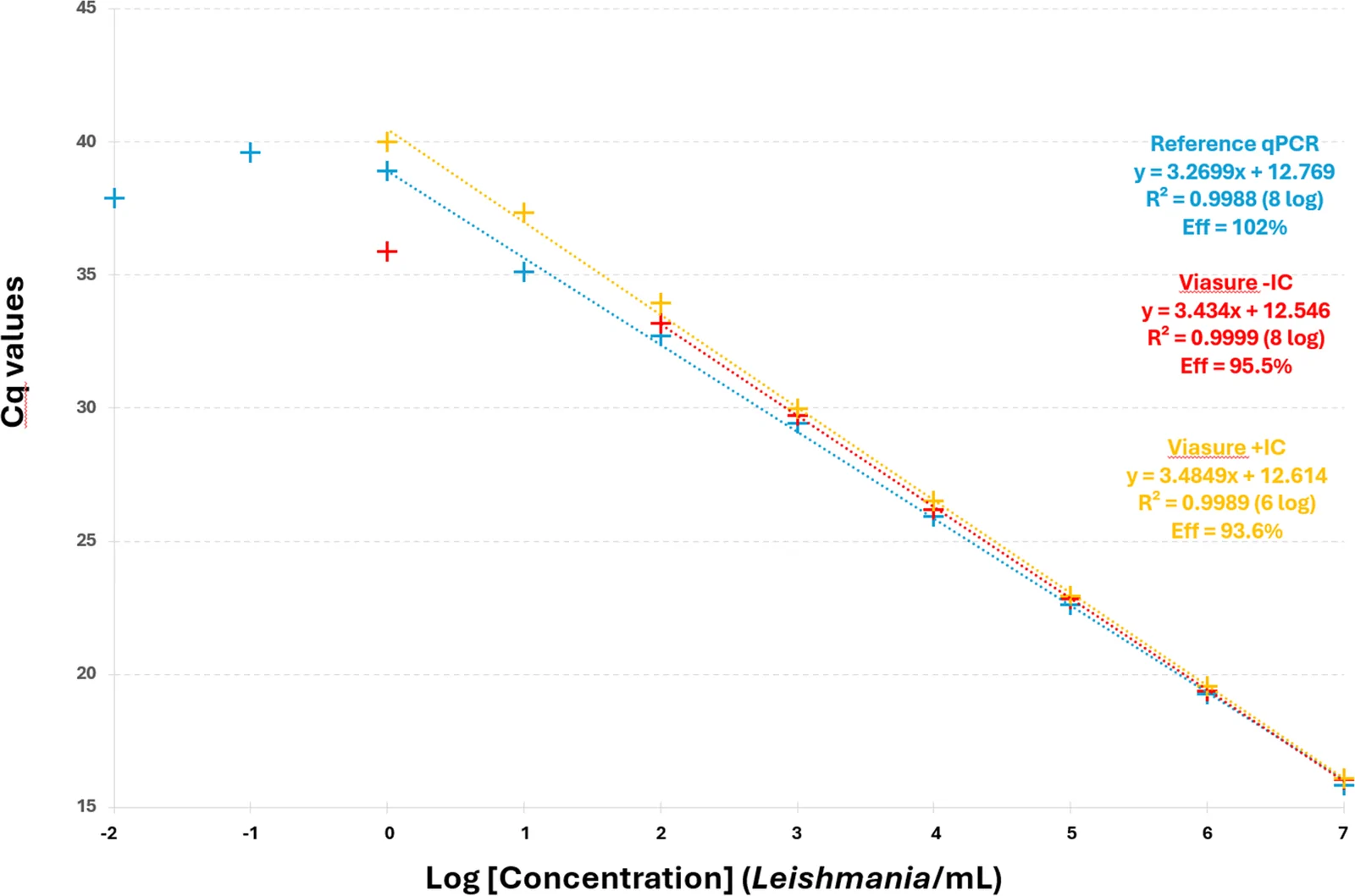
Linearity zone and PCR efficiency (EFF) estimated from the standard curves obtained in the one-tenth serial dilution assays.

**Fig 3:**
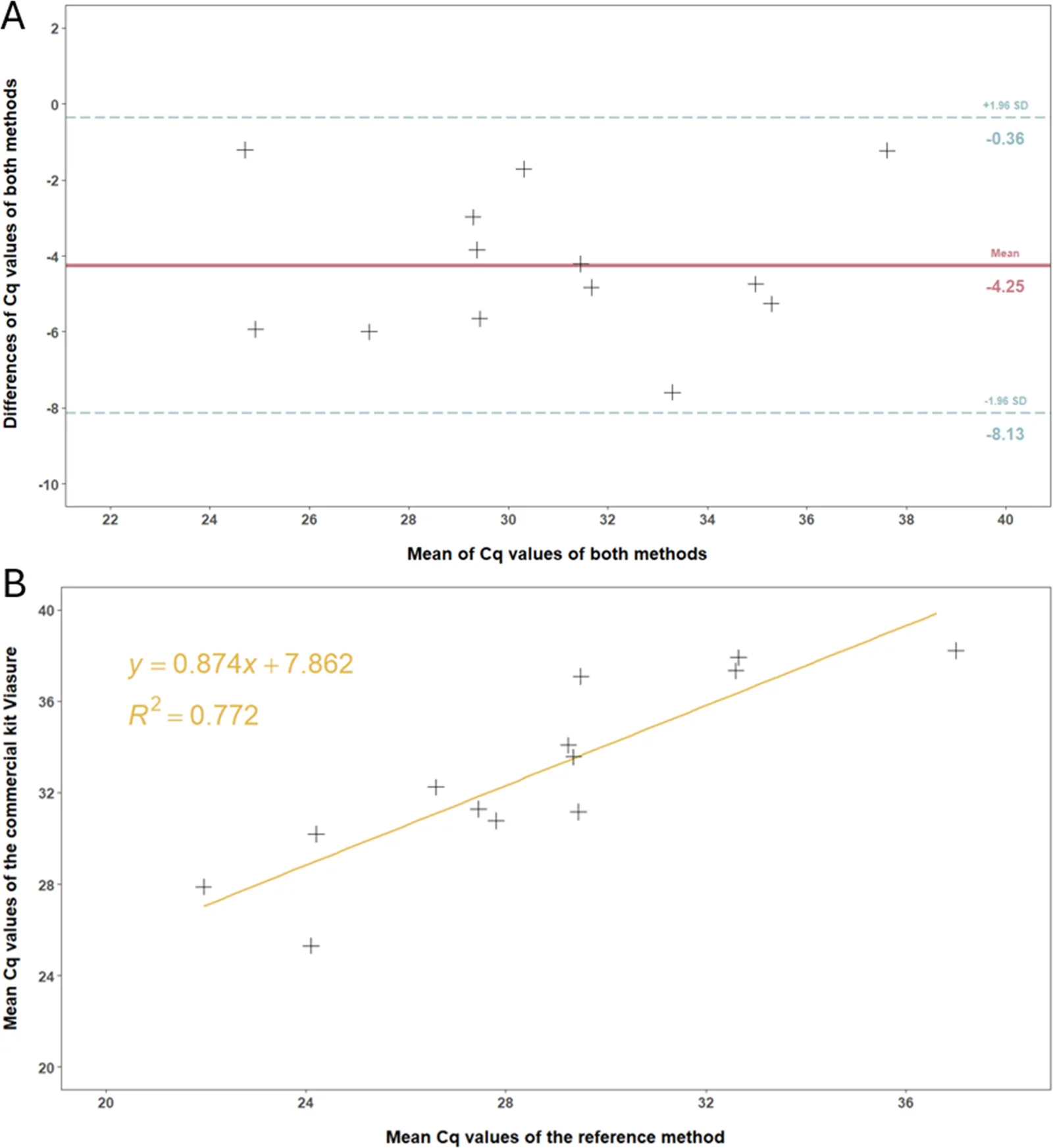
Bland-Altman and Correlation plots. (**A**) Bland-Altman plot comparing the Cq values of the reference method and commercial method. Thirteen samples are represented, as 5 of the 18 samples positive with both methods yielded very late Cq values (37.6–40) with the commercial assay and were excluded because they were close to the limit of detection and outside the assay's linear quantification range. For each sample (black cross), the mean Cq value of the two methods is plotted on the *x*-axis and the difference between the two Cq values on the *y*-axis. The red line represents the mean difference, and the green dotted lines indicate the limits of agreement (mean ± 1.96 SD). (**B**) Correlation plot representing the concordance between the Cq values obtained using the reference method and commercial method for the molecular detection of *L. donovani* complex. The linear regression equation and *R*^2^ value are presented in yellow.

The Viasure kit was initially tested both with and without the internal control (IC) to evaluate whether its inclusion affected analytical performance. Both configurations yielded comparable results, showing the same lowest detectable concentration (1 *Leishmania/*mL), same performance score at 18/32 (56%), similar stringent score and a ΔCq ranging from 0 to 0.7 in the linear zone of the one-tenth serial dilution assay (Table 2). We therefore concluded that the IC does not exert a competitive effect on the target PCR. PCR efficiencies were calculated at 95.5% and 93.6% for the Viasure qPCR without and with IC respectively and 102% for the reference qPCR (Fig. 2). The linearity range was supported by high correlation (*R*^2^ > 0.999), spanning eight logs for the Viasure method and nine logs for the reference method (Table 2; Fig. 3). The total performance score (see Materials and Methods) was 18/32 (56%) for the Viasure method, and 27/32 (84%) for the reference method. The most stringent score corresponds to the three lowest concentrations and was calculated at 2/12 (17%) for the commercial method without IC and 1/12 (8%) for the commercial method with IC and 7/12 (58%) for the reference method (Table 2).

Based on the serial-dilution results, we hypothesized that the Viasure assay would have an LOD_95_ of ~0.5 *Leishmania* per PCR well. To verify this, samples containing 0.25, 0.5, and 1 parasite per well were produced, extracted, and tested in 20 replicates each (Table 3). The Viasure assay produced hit rates of 90% (18/20), 95% (19/20), and 100% (20/20), respectively, while the reference method detected all replicates at every concentration. These data confirm an LOD_95_ of 0.5 parasites per well for the Viasure assay. In contrast, the reference method did not reach its LOD_95_ within this range, indicating an LOD below 0.25 parasites per well and therefore superior sensitivity.

**TABLE 3:** Estimation of the limit of detection of both methods

	1 Leishmania/PCR well			0.5 Leishmania/PCR well			0.25 Leishmania/PCR well		
	Cq value (mean ± SD)	Score	Hit rate	Cq value (mean ± sd)	Score	Hit rate	Cq value (mean ± SD)	Score	Hit rate
In-house qPCR assay developed by Mary et al. (30)	31.0 ± 0.1	20/20	100%	31.5 ± 0.2	20/20	100%	32.1 ± 0.2	20/20	100%
“Leishmania Viasure”	34.6 ± 0.8	20/20	100%	37.0 ± 1.2	19/20	95%	38.7 ± 1.2	18/20	90%

To assess specificity, we evaluated whether the PCR assay could detect DNA from other trypanosomatids (see Materials and Methods). Whereas the in-house qPCR assay developed by Mary et al. (30) yielded negative results for all tested non-target species, the Viasure assay did not detect *Trypanosoma cruzi* DNA but detected *T. brucei* DNA (Table 4).

**TABLE 4:** Specificity assessment of the VIASURE Leishmania assay: no detection of *T. cruzi* DNA but cross-reactivity with *T. brucei* DNA

	*T. cruzi* TGDNA25S-03			*T. cruzi* TGDNA25S-05			*T. brucei*		
	Qualitative result			Qualitative result			Qualitative result		
In-house qPCR assay developed by Mary et al. (30)	Negative			Negative			Negative		

	Qualitative result	Cq value	IC	Qualitative result	Cq value	IC	Qualitative result	Cq value	IC
“Leishmania Viasure”	Negative	NA^*a*^	24.56	Negative	NA	24.47	Positive	23.24	23.47

^*a*^ NA, not applicable.

#### Clinical sensitivity

Thirty blood DNA extracts were analyzed in duplicate using both methods, each incorporating an IC to detect potential PCR inhibition. Assessing DNA extraction quality was performed in the routine practice using a human albumin PCR (see Materials and Methods). Based on the diagnostic criteria outlined in the Materials and Methods section, VL was confirmed in 21 samples and ruled out in 9, as shown in Fig. 1 and Table S1. The qualitative comparison between methods, summarized in the confusion matrix (Table 5), yielded a Cohen’s kappa coefficient of 0.8, indicating substantial agreement based on the scale of Landis and Kock (35). Specifically, 18 samples tested positive by both methods, 9 were consistently negative, and 3 samples showed discordant results. The Cq values obtained in routine ranged from 30 to 36.6 (see Table S1). These discordant cases are false negatives from the Viasure method, given that VL was confirmed in those patients. All discordant samples were reanalyzed in a second experiment with the reference method to validate the findings and eliminate an issue with the preservation of the DNA extract. Additionally, among the 18 concordant positive samples, 5 samples exhibited inconsistent positivity when analyzed with the commercial method (Table S1).

**TABLE 5:** Confusion matrix between the two qPCR methods^*a*^

		Viasure	
		Positive	Negative
In-house qPCR assay developed by Mary et al. (30)	Positive	18^*b*^	3
	Negative	0	9

^*a*^ Confusion matrix summarizing the qualitative results obtained from the case in the clinical cohort. Cohen’s kappa agreement coefficient = 0.8 (substantial agreement).

^*b*^ Among the 18 true positive samples, 5 samples showed inconsistent positivity, indicating intra-sample variability, though they were classified as positive for agreement analysis.

To test the agreement and the concordance between the quantitative results obtained with both methods, a Bland-Altman plot and a correlation plot were performed with the 13 clinical values concordantly positive. The samples found to be inconsistently positive were removed from this analysis. Both methods present good agreement, with no samples outside the ±1.96 standard deviation range in a Bland-Altman plot (Fig. 3) and a moderate correlation with an *R*^2^ calculated at 0.772 (Fig. 3). The difference in Cq values was 4.25 ± 1.98.

## DISCUSSION

Accurate diagnosis of VL is critical due to its clinical overlap with numerous other conditions (8). Serological assays, although widely implemented, suffer from variable sensitivity and specificity, limiting their reliability (7, 10). Parasitological methods, historically considered the gold standard for their high specificity, are constrained by low sensitivity and invasive sampling requirements (10). In contrast, qPCR combines high sensitivity with high specificity and minimal invasiveness, establishing itself as the cornerstone of VL diagnosis (19). Commercial kits are being increasingly adopted in molecular diagnostic laboratories for infectious diseases, with a growing number of options available for VL (Table 1). These kits often offer ready-to-use protocols and practical implementation for standardization, which facilitate the accreditation process of medical microbiology laboratories. However, their performance should not be assumed and must be critically evaluated by independent experts. The Parasitology-Mycology Laboratory at Montpellier University Hospital hosts the French National Reference Center for Leishmaniasis (CNR-L). Each year, approximately 1,000 clinical samples are submitted to the laboratory for the diagnosis of visceral (VL) and cutaneous leishmaniasis (CL). In line with its expertise, the CNR-L has already assessed commercial diagnostic kits for VL (29). To evaluate the Viasure *Leishmania* Real-Time PCR Detection Kit, we determined both analytical and clinical performances, comparing the results obtained with the Viasure qPCR with those obtained using an in-house reference method developed by Mary et al. (30).

Good PCR efficiencies and large linear ranges were found for the Viasure qPCR and the reference method, demonstrating that both methods have been finely optimized. However, when more stringent criteria were applied, it was found that the performance of the reference method was better than that of the Viasure qPCR. Indeed, performance scores of the reference PCR are better (84% vs 56% and 58% vs 12.5%) (see Table 2). In addition, the LOD of the Viasure kit was estimated at 0.5 *Leishmania* per PCR well. For the reference method, the LOD was not reached within the tested concentration range. It was therefore clearly lower than that of the commercial assay. As for clinical sensitivity was estimated on 30 samples, corresponding to 25 patients. All nine negative samples were correctly identified as true negatives by both methods, with no false-positive results. Concerning the 21 VL samples, three samples were found negative using the Viasure qPCR. Notably, these false-negative results were obtained from follow-up specimens collected after treatment initiation, at a stage when the diagnosis had already been established and parasite loads were expected to be lower. The direct clinical impact of these false-negative results was therefore limited in this cohort. Among the 18 concordantly positive samples, 5 samples yielded inconsistent positive signals. This observation underlines the importance of using multiple replicate PCR wells, as circulating parasite loads in patients can be low and close to the detection threshold of the assay (11). Taken together, these results suggest that the Viasure method is less clinically sensitive than the reference method. Similar results were reported elsewhere when the Viasure method was compared with another commercial kit (28). The Viasure method failed to amplify parasites in six clinical samples (one VL and five skin biopsies from cutaneous leishmaniasis) from a cohort of 33 (28). Reduced clinical sensitivity was also observed in a previous study, in which we compared our reference method with the quanty Leishmaniae, Clonit (Ref RT-63q; Launch Diagnostics, Paris, France) qPCR method, which, like the Viasure method, also targets the 18S ribosomal RNA sequences (29). Twenty-seven samples were common to both studies (Table S1).

Undetected or delayed diagnosis of VL can lead to disease progression to severe or fatal stages, particularly since patients rarely have a second opportunity for accurate diagnosis once the initial diagnostic window is missed. Minimal parasitemia is frequently observed in VL and contributes to diagnostic challenges. Given that low-level parasitemia can lead to missed diagnoses with serious or fatal consequences, molecular assays must achieve high analytical sensitivity. High analytical sensitivity is also critical for patient follow-up: qPCR-based monitoring can detect low-level parasitemia post-treatment, which strongly correlates with clinical relapse risk (19).

The choice of genetic target is a critical determinant of sensitivity in molecular assays (19). In our view, the lower sensitivity observed here is largely attributable to the selected DNA target and, more specifically, to differences in the number of available target copies. Indeed, kinetoplast DNA consists of thousands of minicircle copies per parasite, providing an exceptionally high template abundance for amplification; ribosomal DNA occurs in far fewer copies (20–22). This lower copy number limits their utility in contexts where missed diagnoses could have severe consequences.

Regarding the specificity of the Viasure assay, according to the manufacturer’s documentation, *in silico* analysis of primer and probe alignments indicated that the kit is predicted to detect the following species belonging to the subfamily *Leishmaniinae: Crithidia expoeki*, *Crithidia bombi*, *Crithidia brevicula*, *Crithidia mellificae* (without mismatches), and *Lotmaria passim* (with mismatches). The manufacturer states that these species have no clinical relevance for the diagnosis of leishmaniasis. Cross-reactivity testing was performed by the manufacturer against a wide range of pathogens, including *Trypanosoma cruzi*; however, *Trypanosoma brucei* was not evaluated. Here, we confirmed the specificity of the Viasure assay with respect to *T. cruzi*. However, we also demonstrated that the assay can detect *T. brucei* DNA. Consequently, a positive result obtained with this assay should raise the possibility of human African trypanosomiasis as a differential diagnosis, notwithstanding the rarity of the disease and its distinct nosological and epidemiological profile relative to visceral leishmaniasis.

In summary, molecular assays with high analytical sensitivity are indispensable both for initial diagnosis and for monitoring in VL to prevent delayed treatment, disease progression, and relapse. Kinetoplast DNA-targeted qPCR, such as the reference method, should be preferred over ribosomal DNA-based alternatives, including the Viasure kit, where high analytical sensitivity is required, particularly for blood-based diagnosis and monitoring of VL.

## SUPPLEMENTAL MATERIAL

Table S1 jcm.00666-26-s0001.xlsx Raw data.

ASM does not own the copyrights to Supplemental Material that may be linked to, or accessed through, an article. The authors have granted ASM a non-exclusive, world-wide license to publish the Supplemental Material files. Please contact the corresponding author directly for reuse.
